# Cost-effectiveness of myopia-control spectacles and contact lenses for children and adolescents in Wales

**DOI:** 10.1186/s12962-025-00632-w

**Published:** 2025-06-04

**Authors:** Hayley Bennett, Andy Britton, David O’Sullivan, Francesca Lado

**Affiliations:** 1 Health Technology Wales, Cardiff, Wales; 2 Optometry Wales, Haverfordwest, Wales; 3 Welsh Government, Cardiff, Wales; 4 NHS Wales, Llanfairfechan, Wales

**Keywords:** Cost effectiveness, Myopia, Near-sightedness, Optical devices, Contact lenses, Orthokeratology

## Abstract

**Background:**

Early intervention to slow childhood progression of myopia may improve quality of life and prevent future complications that burden individuals and healthcare systems. This study assessed the cost-effectiveness of myopia-control spectacles and contact lenses for the reduction of myopia progression among children and adolescents in Wales.

**Methods:**

A cost-utility analysis compared peripheral plus spectacle lenses (PPSL), multifocal soft contact lenses (MFSCL) and orthokeratology against single-vision correction. Efficacy and safety were informed by a Cochrane systemic review and meta-analyses. Quality-adjusted life years (QALYs) and costs incurred by NHS Wales were modelled over a lifetime horizon and discounted at 3.5%. Sensitivity analyses estimated uncertainty in incremental cost-effectiveness ratios (ICERs).

**Results:**

PPSL was estimated to provide minimal benefit at a higher cost than single-vision correction. MFSCL gave a 0.28 QALY improvement at an additional cost of £4,040; corresponding to an ICER of £8,367 versus single-vision correction. Orthokeratology provided 0.5 QALYs at an additional cost of £3,732; corresponding to an ICER of £3,995 versus single-vision correction. In probabilistic sensitivity analysis, ICERs were below £20,000 in 71% and 90% of simulations for MFSCL and orthokeratology, respectively. Orthokeratology was the most cost-effective strategy in 76% of simulations. Cost-effectiveness was influenced by changes in progression rates, intervention costs and the utility of high myopia. However, orthokeratology remained the most cost-effective strategy throughout.

**Conclusions:**

MFSCL and orthokeratology may be cost-effective options to slow the progression of myopia at thresholds applied in the UK. Further research is needed to understand the long-term effects of myopia-control interventions and their impact on quality of life.

**Supplementary Information:**

The online version contains supplementary material available at 10.1186/s12962-025-00632-w.

## Introduction

Myopia, also known as short-sightedness or near-sightedness, is a refractive anomaly of the eye that causes distant objects to appear blurred, while closer objects appear clearly. Myopia typically develops during childhood and can result from the eyeball growing too long or the cornea or lens being too curved. The prevalence of myopia varies by region, ethnic group and age, noticeably increasing after the age of six years [1, 2]. Some children are more likely to develop myopia because of genetic factors, but sociological and lifestyle factors might also contribute to the increasing prevalence of myopia globally [3, 4].

Children with myopia may experience difficulties in school, reduced ability to participate in sports, and social exclusion. Myopia negatively impacts quality of life [5] and high levels may be a limiting factor for jobs with minimum sight requirements. The progression of myopia is associated with increased risks of retinal detachment, macular degeneration, cataracts and glaucoma, and can lead to significant visual impairment [6]. A growing body of evidence shows that people with any level of myopia may be at excess risk of complications [7]. Analysis of data from large population studies suggests that slowing myopia progression by one dioptre (D) could reduce the chance of developing myopic maculopathy by 40%, irrespective of initial refractive error [8]. Interventions that slow the progression of myopia therefore have the potential to lower the burden of myopia and related complications for both individuals and the National Health Service (NHS).

Current standard care in Wales is to correct vision with single-vision spectacles or contact lenses. Free sight tests and vouchers towards the cost of glasses or contact lenses are available for children under the age of 16 and for 16 to 18-year-olds in full-time education. There is no provision under the NHS for myopia-control interventions which aim to slow the progression of myopia, and any prescribed interventions are paid for privately.

The World Council of Optometry recommends that standard care for myopia should include both correction and evidence-based interventions to slow its progression [9]. UK guidance from the College of Optometrists recommends that optometrists should offer myopia-control spectacles and contact lenses if they have the relevant expertise or sign-post to practices that do [10]. This means that optometrists have a duty of care to have appropriate discussions about the provision of myopia management, in addition to advice on prevention.

Myopia-control interventions are only available privately in Wales and are more expensive than single-vision correction. The higher cost and potential need to travel for these services means that myopia-control interventions are not accessible to all families in Wales, and could leave some children at higher risk of developing complications.

Healthy Technology Wales (HTW) is a national body that assesses non-medicine health and care technologies and produces guidance on their use in Wales. HTW reviewed clinical and cost-effectiveness evidence published before April 2023, during its health technology assessment of myopia-control spectacles and contact lenses [11]. One previous economic study was identified that compared the costs of myopia-control and correction strategies in Australia and China [12]; however, none were identified that assessed cost-effectiveness or considered a UK setting.

To address this gap in the evidence, the objective of this analysis was to estimate the cost-effectiveness of myopia-control spectacles and contact lenses compared with standard care for children and adolescents in Wales.

## Methods

A cost-utility analysis was undertaken to compare management strategies for myopia among children and adolescents from the perspective of the NHS in Wales. The cohort profile was approximated from studies included in a recent systematic review and meta-analyses by Lawrenson et al. [13]; 52% of modelled children were female, with a mean age of 10.4 years and spherical equivalent refractive error (SER) of -2.70 D at baseline.

### Comparators

We compared the following optical myopia-control or correction strategies within a fully-incremental analytical framework:

- Single-vision lenses (SVL): current standard care.
- Peripheral plus spectacle lenses (PPSL).
- Multifocal soft contact lenses (MFSCL).
- Orthokeratology.

Single-vision spectacles and contact lenses were grouped together to reflect standard care in Wales and reporting from Lawrenson et al. [13]. Though children eligible for MFSCL or orthokeratology may be more likely to use single-vision contact lenses under current practice. Lawrenson et al. [13] found neither rigid gas-permeable contact lenses or multifocal spectacle lenses slowed myopia progression to a clinically significant degree, so these strategies were not modelled. Pharmaceutical interventions, such as atropine eye drops, alone or in combination with optical interventions, were outside the scope of the HTW appraisal.

### Model structure and assumptions

**Fig. 1:**
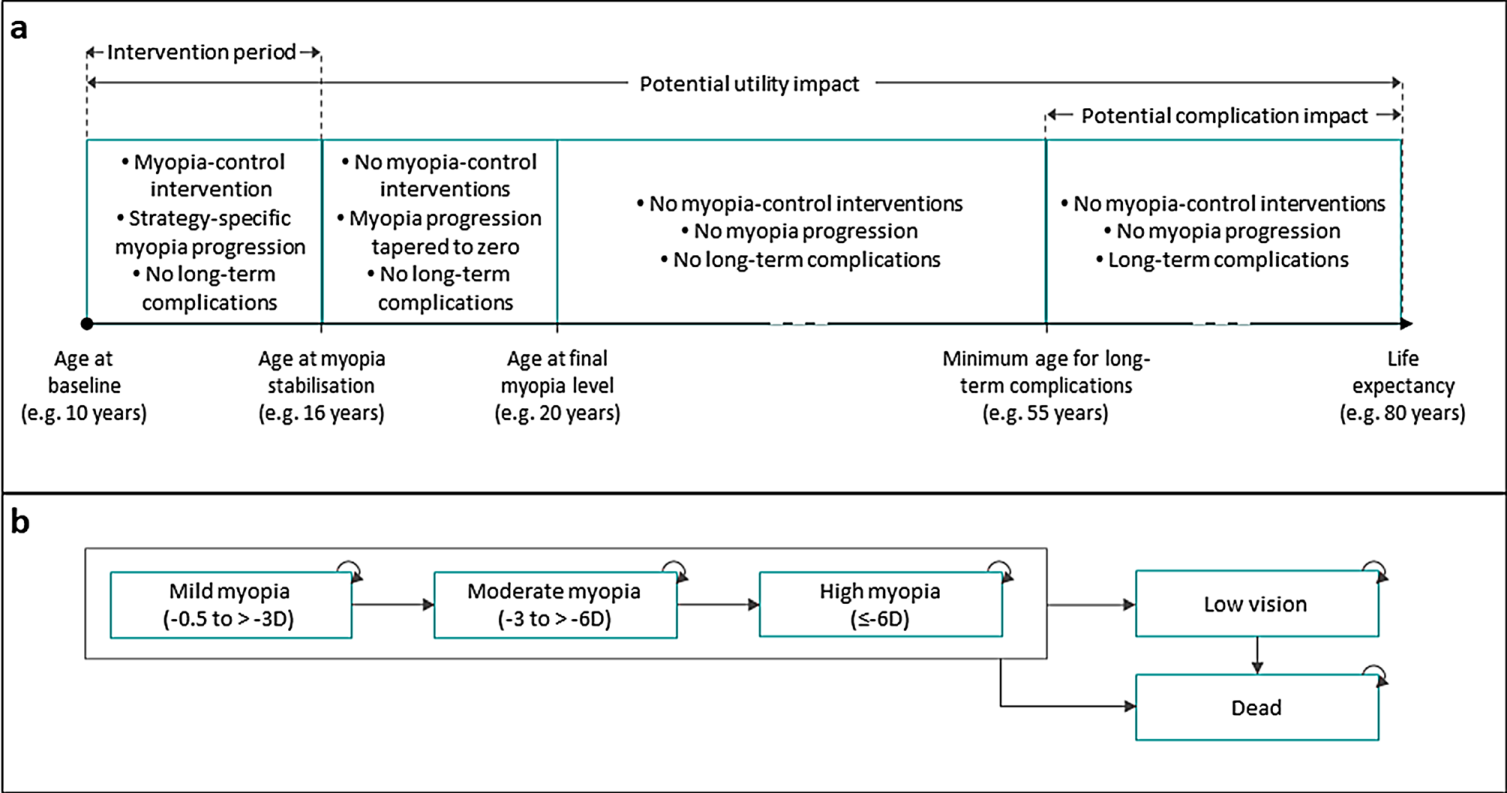
Overview of model structure (**a**) and health states **(b)**; Figure depicts the maximum intervention period but the modelled duration was specific to each strategy

A cohort-level state-transition model was developed in Microsoft Excel to estimate myopia progression in terms of SER and the incidence of long-term eye-related complications in later adulthood (Fig. 1). Myopia severity was defined by SER level, with thresholds of -3 D and − 6 D corresponding to moderate and high myopia, respectively [14]. Health-related quality of life was modelled according to myopia severity. Myopic macular degeneration, retinal detachment, cataract, glaucoma and progression to a low-vision health state were modelled from age 55 years using published rates, adjusted for SER level [7, 12, 15]. Baseline SER was sampled to model variation in starting values and the impact of myopia-control strategies was captured via reduced rates of SER progression.

Lifetime direct healthcare costs and quality-adjusted life years (QALYs) were evaluated over six-monthly cycles and discounted at 3.5% annually. We estimated incremental cost-effectiveness ratios (ICERs), which represent the extra cost of a strategy divided by the additional QALYs it provides. We also estimated total health benefit, which combines the QALYs estimated for a strategy with the potential QALYs foregone elsewhere by paying for that strategy. ICERs up to £20,000 per QALY were considered cost-effective [16]. The strategy with the highest health benefit at this threshold was considered the best value for money (referred to as optimal). Further explanation of these concepts is available in the HTW Appraisal Process Guide [16].

Full detail of the inputs and assumptions used in the analyses are provided in Tables A1, A2 and A3 (Additional File 1). Key modelling assumptions included:

- Unobserved effects of orthokeratology on SER were modelled from effects on axial length.
- Rates of SER progression modelled in year two were maintained in subsequent years up to the age of around 16 years, when progression slowed, before stopping at age 20 years.
- Myopia-control interventions ended at age 16 years to coincide with the slowing of myopia progression.
- There was no rebound in SER levels after the use of myopia-control interventions ended.
- World Health Organisation (WHO) disability weights could be used to estimate the impact of myopia of health-related utility.

Feedback from ten UK optometry experts informed the final model design and analysis. No responses were received from organisations contacted as part of HTW’s patient and public involvement process. More information on these processes is available [11, 16].

### Efficacy and safety

Model inputs describing the efficacy and safety of each strategy were informed by the Cochrane review by Lawrenson et al. [13] (Table 1). The networks underlying the authors’ network meta-analysis (NMA) were poorly connected and its estimates were no more precise than direct estimates from meta-analysis. We therefore applied pooled estimates from meta-analyses in our base case and used the NMA values in sensitivity analysis. In the base case, PPSL was stopped after two years because no statistically significant effect was reported at this time point. We conservatively assumed that ineffective methods would be discontinued, though this may be difficult to determine in practice.

SER effect estimates were not available for orthokeratology because these lenses temporarily reshape the cornea, which means SER progression can only be assessed after lens wear has ended. We followed the modelling approach of Fricke et al. [12] and assumed that orthokeratology had the same proportional effect on SER as axial length. Though this approach is associated with considerable uncertainty, it is supported by published correlations between progression of SER and axial length which were used in sensitivity analysis [17, 18].

Lawrenson et al. [13] reported that adverse events for MFSCL and PPSL were generally mild with similar rates to SVL, but were more common with orthokeratology. We therefore modelled discontinuation due to adverse events in the first cycle of orthokeratology use only.

**Table 1:** Base case strategy effects, safety and cost (2023 £) inputs

Strategy	SVL	PPSL	MFSCL	Orthokeratology
SER change from baseline to year 1	-0.65 D	N/A	N/A	N/A
SER change from baseline to year 2	-1.02 D	N/A	N/A	N/A
SER difference v SVL at year 1	N/A	0.51 D	0.26 D	0.40 D^a^
SER difference v SVL at year 2	N/A	0.00 D	0.30 D	0.51 D^a^
Discontinuation due to adverse events	0%	0%	0%	5%
Initiation cost	N/A	N/A	£100	£200
Monthly cost	N/A	£44	£40	£40
WGOS sight test cost^b^	£43	N/A	N/A	N/A
WGOS voucher cost (subject to SER)^b^	£22 (> -6 D) £42 (≤ -6 D)	N/A	£22 (> -6 D) £42 (≤ -6 D)	N/A

^a^Orthokeratology assumed to have the same proportional effect on SER as axial length, with an estimated 61% and 50% reduction in elongation at years 1 and 2, respectively

^b^Costs incurred annually under the age of 16 years, then every two years for 16 to 18 year olds in full time education

Abbreviations: SER, spherical equivalent refractive error; SVL, single-vision lenses; WGOS, Wales General Ophthalmic Service

### Healthcare resource use, costs and health-related utility

Modelled costs included myopia management, optometry visits, and management of complications. For SVL, only the costs of sight tests and correction provided up to the age of 18 under the Wales General Ophthalmic Service contract were included; out-of-pocket expenses incurred by people with myopia and their families were not included. Myopia-control intervention costs were estimated from the websites of private opticians, who commonly offer monthly payment plans after an initial instalment. We identified costs for different products across 10 providers, used a mid-range option as our base case, and tested upper and lower cost profiles to reflect the wide range of published prices. Other health-state and complication costs were obtained from the British National Formulary (BNF), NHS Reference Costs (2020/21) and literature [19–22]. Costs were inflated to 2021/22 prices where necessary [23].

QALYs were estimated by combining life year estimates with an age-adjusted baseline utility [24] and utility decrements, applied additively. In the absence of data characterising the impact of myopia on health-related utility, we followed the assumptions of Hong et al. [25] to apply WHO disability weights to myopia health states, updated with the latest estimates [26]. Utility decrements associated with long-term complications were sourced from the literature [27, 28]. We did not model any utility differences for the use of spectacles versus contact lenses, or the reduced need to correct vision during the daytime with orthokeratology. Limited evidence was identified on how alternative myopia-control or correction strategies might impact a child’s quality of life.

### Sensitivity and scenario analyses

We used scenario analyses to test the impact of alternative modelling assumptions and inputs on results. Deterministic sensitivity analysis was used to assess the relative influence of each input parameter (± 20% of base case value) and determine the key drivers of model estimates. Probabilistic sensitivity analysis was performed to assess combined parameter uncertainty by modelling 1,000 cohorts of 1,000 children, with model input values drawn independently from specified parameter distributions.

### Budget impact analysis

Following the publication of guidance from the HTW Appraisal Panel [11], we estimated the potential impact of introducing orthokeratology to Welsh healthcare budgets at the national level. We modelled the prevalence of myopia among children aged at least six and less than 16 years using estimates from elsewhere in the UK [1, 29]. Costs were informed by the cost-utility model. Data were not available to support a detailed analysis considering the distribution of myopia levels or predicted uptake of myopia-control interventions across Wales.

## Results

### Base case analysis

All myopia-control strategies were estimated to increase costs compared with SVL and all but PPSL provided small QALY gains (Table 2). PPSL was estimated to provide minimal benefit because its modelled use was ended after two years, with no statistically significant SER advantage over SVL. The higher intervention costs of MFSCL and orthokeratology were partially offset by the avoidance of more expensive optical vouchers and long-term complications, including entering the low-vision health state. Estimated QALY gains were driven by the avoidance of progression to high myopia for some children. Cost-effectiveness estimates improved over longer modelled time horizons, as more of these QALY gains were captured.

Over a lifetime horizon, both MFSCL and orthokeratology were estimated to be cost-effective compared with SVL with ICERs well below the £20,000 threshold of £8,367 and £3,995 per QALY, respectively. In fully incremental analysis, orthokeratology was estimated to represent the best value for money of the strategies considered. Orthokeratology was both cheaper and more effective than MFSCL in this analysis.

**Table 2:** Cost-effectiveness results

Strategy	SVL	PPSL	MFSCL	Orthokeratology
**Base case analysis:**				
Total costs	£1,718	£2,628	£4,040	£3,732
Total QALYs	22.91	22.91	23.18	23.41
Total health benefit	22.82	22.78	22.98	23.22
Incremental costs v SVL	N/A	£910	£2,322	£2,014
Incremental QALYs v SVL	N/A	0.00	0.28	0.50
Incremental net health benefit v SVL	N/A	-0.04	0.16	0.40
ICER (£/QALY) v SVL	N/A	> £1 M	£8,367	£3,995
Comparator in fully-incremental analysis	Reference	N/A	N/A	SVL
**Probabilistic sensitivity analysis:**				
Cost-effective v SVL (%)	N/A	0%	71%	90%
Optimal strategy (%)	5%	0%	18%	76%

Abbreviations: ICER, incremental cost-effectiveness ratio; M, million; MFSCL, multifocal soft contact lenses; N/A, not applicable; PPSL, peripheral plus spectacle lenses; QALY, quality-adjusted life-year; SVL, single-vision lenses; v, versus

### Sensitivity and scenario analyses

**Fig. 2:**
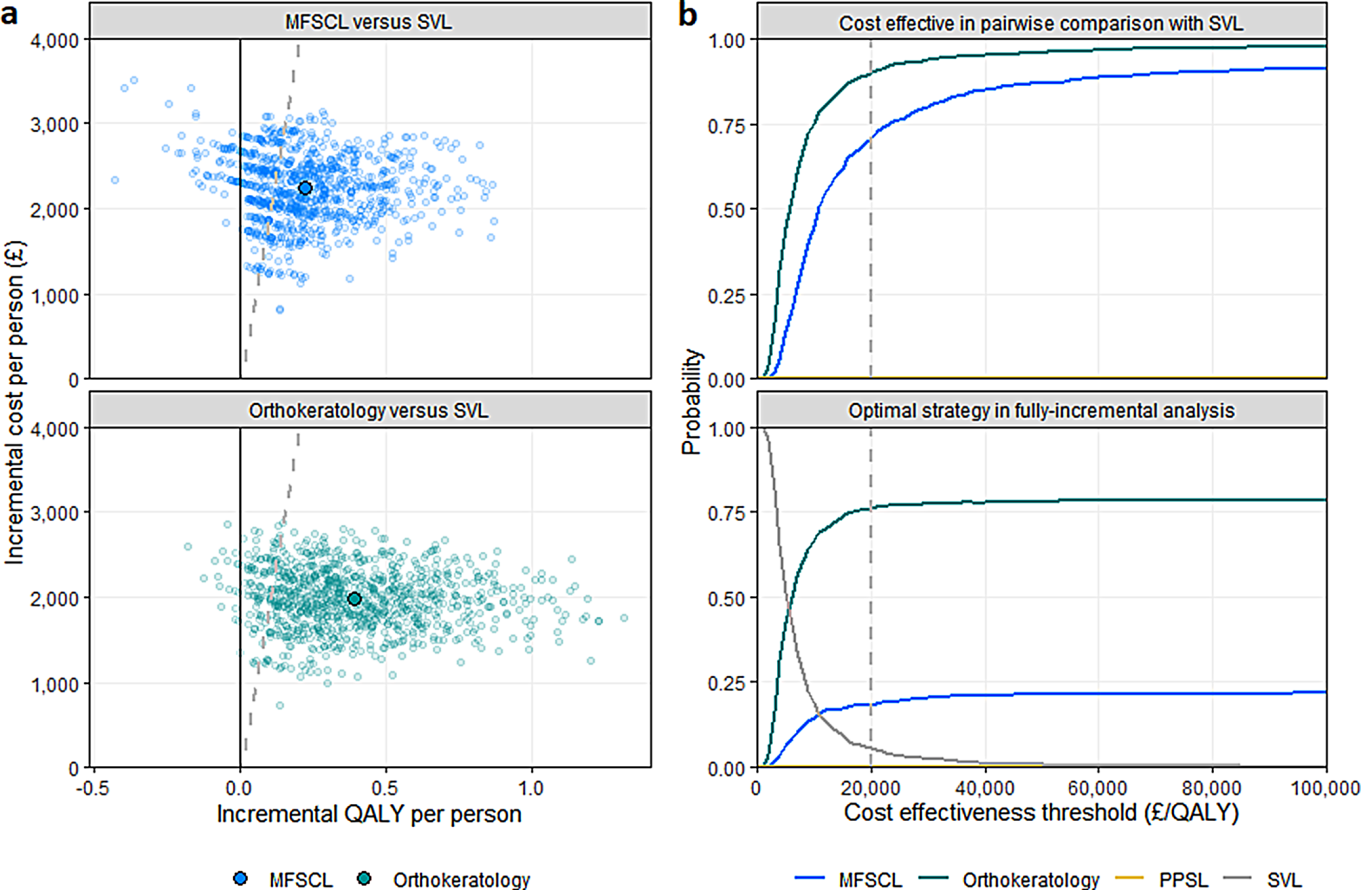
Results of probabilistic sensitivity analysis shown on the cost-effectiveness plane (**a**) and as cost-effectiveness acceptability curves (**b**)

In probabilistic sensitivity analysis (Fig. 2), MFSCL and orthokeratology were estimated to be cost-effective compared with SVL in 71% and 90% of iterations, respectively. Orthokeratology was estimated to represent the best value for money among the strategies considered in 76%.

Deterministic sensitivity analysis showed that in addition to the discount rates applied to costs and QALYs, cost-effectiveness results were most sensitive to changes in parameters controlling SER progression, strategy costs and duration, and the impact of high myopia on quality of life (Fig. A1, Additional File 1).

MFSCL and orthokeratology were estimated to be most cost-effective for children with moderate levels of myopia at baseline who could avoid progression to high myopia with intervention. They were less cost-effective for children who were unlikely to progress to high myopia when using SVL, or those who already had high myopia at baseline.

The conclusions of the base case remained largely unchanged in scenario analysis, with three exceptions (Fig. A1 and Table A2 in Additional File 1). MFSCL was not estimated to be cost-effective compared with SVL when alternative utility values were applied. The ICER for MFSCL versus SVL increased to £23,681 when older disability weights [30] were used and to £43,009 when the utility decrements applied to moderate and high myopia were assumed equal. PPSL was estimated to be cost-effective compared with SVL (ICER: £6,334) when NMA estimates from Lawrenson et al. [13] were applied and extrapolated up to age 16, as for the other myopia-control interventions. However, orthokeratology was estimated to provide the best value for money throughout all scenarios tested.

**Fig. 3:**
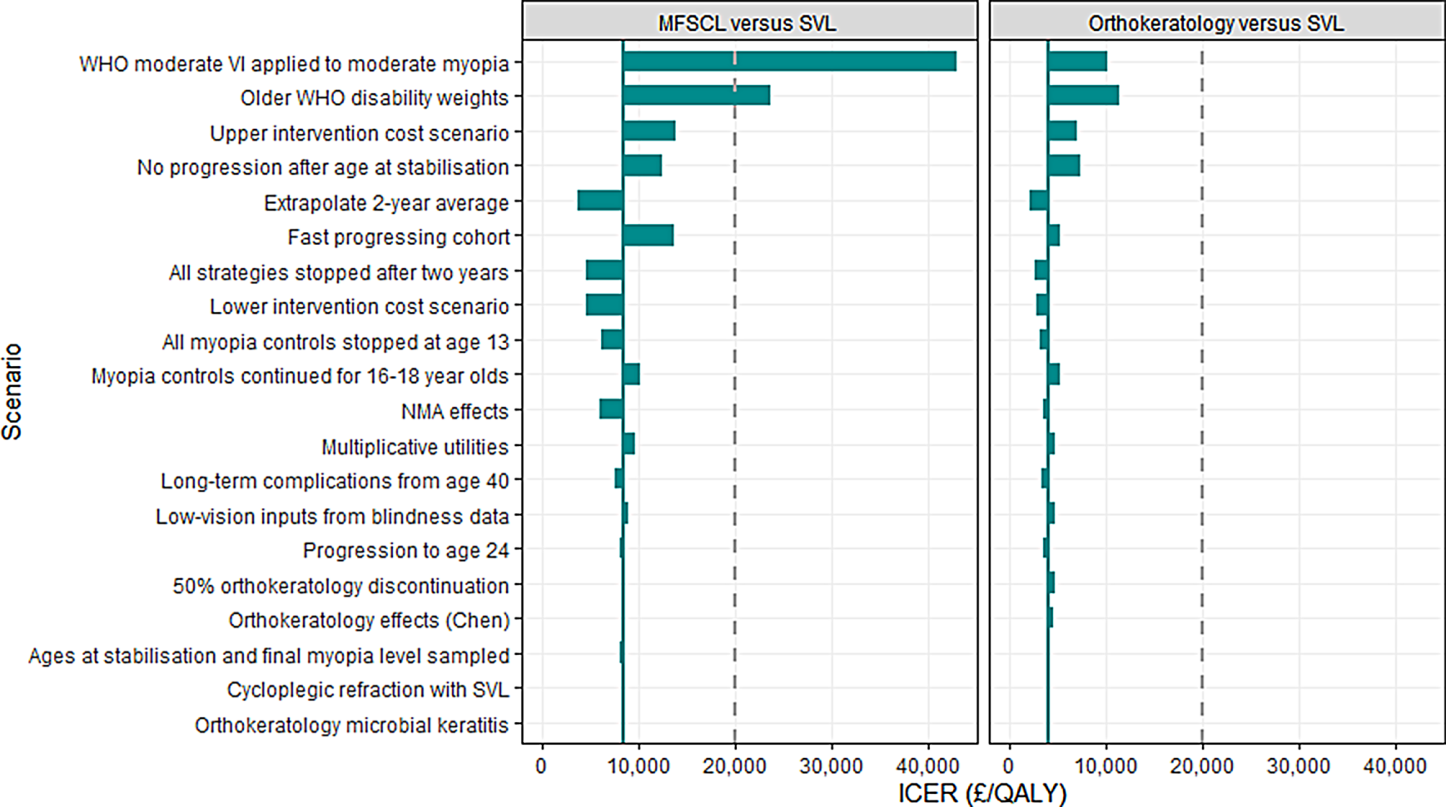
Tornado plots summarising results of 20 most influential scenario analyses

### Budget impact

We estimated that over 29,000 children aged six to 15 might have myopia in Wales (Table A3 in Additional File 1). Provision of orthokeratology for these children was estimated to cost around £18 million more than SVL in the first year (Table 3). One-year budget impact estimates ranged from £13.5 million to £30.9 million using the lower and upper cost profiles applied in scenario analyses. We estimated that an extra 5,885 children may turn six or experience newly onset myopia in subsequent years. Over a five-year horizon, the provision of orthokeratology for all children aged six to 15 years was estimated to have a discounted budget impact of £66.9 (range: £48.7 to £109.9) million compared with current standard care.

**Fig. 4:**
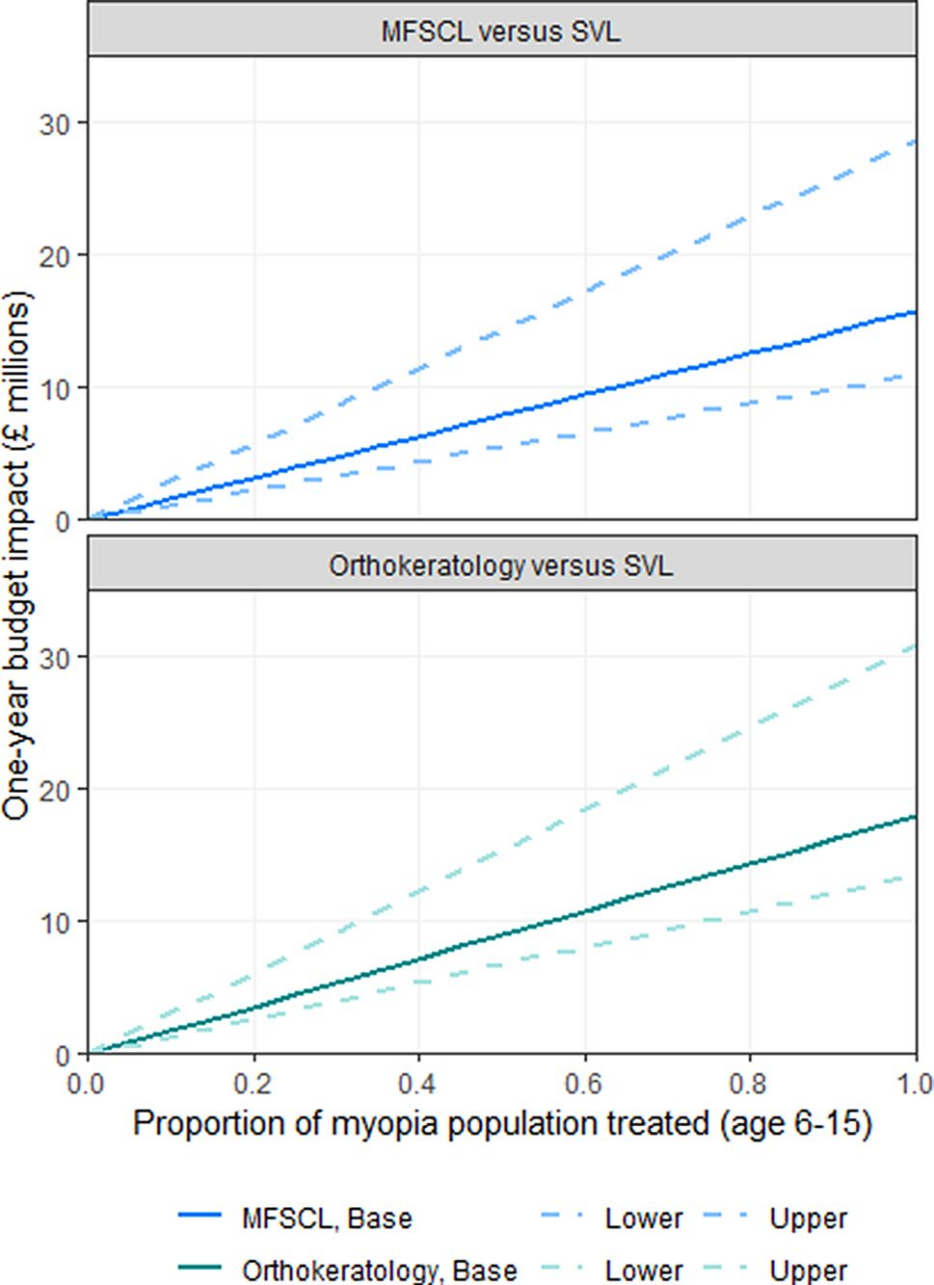
One-year budget impact estimates for varied uptake of orthokeratology or MFSCL

This analysis will overestimate the true budget impact of introducing myopia-control interventions in Wales because it assumes that all children with myopia would use orthokeratology, none would discontinue due to adverse events or for other reasons and the higher cost of SVL for children with high myopia was not captured. In reality, orthokeratology is not suitable for all children and many do not tolerate this type of intervention. Figure 4 shows budget impact estimates for a lower uptake of orthokeratology, with similar results estimated for MFSCL over one year.

**Table 3:** Estimated budget impact of introducing orthokeratology for all children aged 6 to 15 years in Wales

Year	1	2	3	4	5	Total
Children starting strategy	29,246	5,885	5,885	5,885	5,885	52,786
Discounted costs: SVL	£1.9 M	£1.8 M	£1.8 M	£1.7 M	£1.7 M	£8.9 M
Discounted costs: orthokeratology	£19.9 M	£14.7 M	£14.2 M	£13.7 M	£13.3 M	£75.8 M
Budget impact	£18.0 M	£12.9 M	£12.4 M	£12.0 M	£11.6 M	£66.9 M

Abbreviations: M, million; SVL, single-vision lenses

## Discussion

This study showed that from the perspective of the NHS in Wales, MFSCL and orthokeratology may be cost-effective strategies to reduce myopia progression compared with correction alone. Orthokeratology was estimated to provide the best value for money of the strategies considered.

Newer optical interventions, such as diffusion optics technology lenses, and use of pharmacological agents, such as atropine, were not evaluated. These interventions show potential for slowing myopia progression [13, 31] and may become more relevant to Welsh practice if longer-term data are gathered and capacity for independent prescribing increases. Though atropine is not currently licensed or available in the UK for myopia control. Increased availability of evidence-based interventions to manage myopia progression will enable practitioners to tailor strategies to children’s needs and any barriers to care.

To our knowledge, no previous economic evaluations of myopia-control lenses have been published in the UK. Comparison of our findings to non-UK studies is difficult because of differences in healthcare systems and perspectives taken. Typical of UK health technology assessments we took an NHS perspective [16], which excluded out-of-pocket payments for glasses or contact lenses made by families and other costs considered from a societal perspective like travel or lost productivity. Despite this, our findings for MFSCL and orthokeratology are consistent with other studies which report intervention to be cost-incurring versus SVL from a societal perspective in Hong Kong, China and Australia [12, 32]. Comparison of findings for PPSL is further complicated by differences in interventions studied. Fricke et al. estimated that a broader group of myopia-control spectacles, including bifocals, could be cost-saving from a societal perspective in China and Australia [12]. While two cost-utility analyses published since HTW’s review [11] estimated that Defocus Incorporated Multiple Segments (DIMS) spectacles were cost-incurring but cost-effective versus SVL from a societal perspective in Hong Kong [33, 34]. A key difference between these studies and our PPSL analysis is the modelled longevity of effectiveness. As for other forms of myopia control, the long-term effectiveness of PPSL is uncertain. The meta-analysis by Lawrenson et al. showed insufficient evidence of a statistically significant SER effect for PPSL after one year, though one trial of DIMS included in the meta-analysis did show significant benefits over two years [13]. Our scenario analysis showed that modelling assumptions around the long-term effectiveness of PPSL are highly influential to cost-effectiveness conclusions for this intervention.

As with all economic evaluations, there are limitations to our analysis which lead to uncertainty in its estimates. Short-term efficacy data were applied over a longer period and all statistically significant effects were modelled, regardless of effect size. Guiding principles of myopia control clinical trial design from the International Myopia Institute recommend a minimum of three years’ follow-up to assess the efficacy of myopia-control interventions [35]. We extrapolated progression rates from the second year only, to account for the possibility that most of the effect happens in the first year of treatment. However, this approach could still overestimate the effectiveness of myopia-control interventions if it diminishes further with longer-term use. By using estimates from meta-analysis, we did not consider differences between technologies within the same group (e.g. different MFSCL or PPSL designs) or account for differences between clinical trials.

Axial length and SER are both important measures of effectiveness and myopia progression. However, SER was used to model progression and complication risks because the published evidence base was more developed. This required assumptions to map axial length effects for orthokeratology to SER. We modelled relationships between myopia and long-term complications from observations in the absence of myopia-control interventions. Whether the effect of myopia-control interventions on SER and axial length translates to prevention of these complications cannot be proven until they have been in use for a number of decades, because of the delay between childhood myopia and later vision-related complications.

The available effectiveness evidence did not support a full exploration of how cost-effectiveness might vary between children. While the prevention of myopia progression might best be achieved through early intervention, it is still unclear how the effectiveness of myopia-control interventions might differ for different ages, levels of myopia or rates of progression.

At the time of analysis, myopia-control interventions were only available privately in the UK and prices varied considerably between private opticians. Our analysis did not explicitly consider the cost of training or specialist equipment needed to offer myopia-control interventions because providers were expected to have accounted for these within their prices. As no specialist equipment is needed to provide MFSCL, most practices should be able to offer this option without significant investment or organisational change.

A simplified modelling approach was taken for adverse events and discontinuation because of inconsistency and limitations of reporting from clinical trials. No studies were identified prior to analysis that characterised the impact of myopia on health-related utility. When quality of life is captured by myopia studies, vision-specific instruments such as NEI-VFQ are typically used. Consequently, our reliance on assumptions to link myopia progression to utility decrements is associated with considerable uncertainty. It is unknown how well the use of disability weights corresponds to the true impact of myopia in countries where correction is widely available. The utility decrement applied to high myopia (0.089) does appear consistent with recent cross-sectional data from China showing worse scores on the Pediatric Quality of Life Inventory for children with SER no better than − 5 D than those with naturally clear vision (88.04 versus 96.74, out of 100) [36]. The study concluded that high levels of myopia in children can also negatively impact quality of life for parents. This was not captured in our analysis. Finally, the evidence available to inform the analysis came primarily from outside the UK. It is unknown how well this data generalises to Wales.

Comprehensive sensitivity analyses showed the cost-effectiveness conclusions for MFSCL and orthokeratology were largely unchanged when alternative inputs and assumptions were tested. However, these limitations of the available evidence highlight key areas for future research. UK data on the impact of myopia on children’s quality of life and comparative evidence on the long-term progression of myopia under different methods of myopia control would be valuable for future economic evaluations and guidance for clinical practice. As would further evidence on which children are likely to benefit most from intervention.

Myopia has been labelled as a pandemic and is becoming more common globally. Children are developing myopia at younger ages [1] and by 2050 it is estimated that half of the world’s population will be myopic [37], making it an important public health issue.

Spending more time outdoors may delay or even prevent the onset of myopia for some children. Environmental interventions focussed on time outdoors or near-vision activities have an important role to play and UK guidance recommends optometrists encourage a healthy balance for children whether or not myopia management is offered [10].

For children who do develop myopia, barriers to the uptake of myopia-control interventions may include costs and accessibility of services, lack of awareness among families, and concerns around contact lens wear. Appropriate education on the risks of myopia and proper use of interventions is important, particularly for parents and carers who do not have myopia themselves.

## Conclusions

The analysis suggests that MFSCL and orthokeratology may be cost-effective options to slow the progression of myopia at cost-effectiveness thresholds conventionally applied in Wales and elsewhere in the UK. The cost-effectiveness of PPSL remains uncertain. Further research is needed to understand the long-term effectiveness of myopia-control spectacles and contact lenses and their impact on health-related quality of life.

## Electronic supplementary material

Below is the link to the electronic supplementary material.

Supplementary Material 1: Additional File 1 (Additional-file-1.docx) includes detailed model inputs and a summary of deterministic sensitivity and scenario analysis

## Data Availability

All data analysed or generated during this study are included in this article and its additional material.

## Abbreviations

BNF: British National Formulary
D: Dioptre
HTW: Healthy Technology Wales
ICER: Incremental Cost-Effectiveness Ratio
MFSCL: Multifocal Soft Contact Lenses
NHS: National Health Service
NMA: Network Meta-Analysis
PPSL: Peripheral Plus Spectacle Lenses
QALY: Quality-Adjusted Life Year
SER: Spherical Equivalent Refractive Error
SVL: Single-Vision Lenses
WHO: World Health Organisation
