# Supplementary material for: Cost-effectiveness of myopia-control spectacles and contact lenses for children and adolescents in Wales
Source: Cost Eff Resour Alloc. 2025 Jun 4;23:26. doi: 10.1186/s12962-025-00632-w (PMC12139160; doi:10.1186/s12962-025-00632-w)
Supplement: Supplementary file 1 — Supplementary Material 1: Additional File 1 (Additional-file-1.docx) includes detailed model inputs and a summary of deterministic sensitivity and scenario analysis [file 12962_2025_632_MOESM1_ESM.docx]

Additional file 1

Table A1: Full list of model inputs used in base case and probabilistic sensitivity analysis

| **Input** | **Mean** | **Sampling parameters SE or α, β; distribution** | **Source and derivation** |
| --- | --- | --- | --- |
| Male | 48% | 5,576, 6,040; beta | Lawrenson et al. (2023)[1] |
| Age at baseline (years) | 10.4 | 1.1; normal | Lawrenson et al. (2023)[1]; SE estimated from study means. |
| SER at baseline (D) | -2.70 | 1.2; normal | Approximated from characteristics of studies in Lawrenson et al. (2023)[1] meta-analyses. |
| SER change from baseline (D) |  |  | Lawrenson et al. (2023)[1] |
| SVL, year 1 | -0.65 | N/A |  |
| SVL, year 2 | -1.02 | N/A |  |
| SER difference v SVL at year 1 (D) |  |  |  |
| PPSL | 0.51 | 0.15; normal | Lawrenson et al. (2023)[1] meta-analysis |
| MFSCL | 0.26 | 0.05; normal | Lawrenson et al. (2023)[1] meta-analysis |
| Orthokeratology | 0.40 | 0.04; normal | SER effect assumed proportionally equal to axial length effect; 61% reduction estimated from 0.31 mm change from baseline to year 1 with SVL and mean difference of -0.19 mm for orthokeratology versus SVL reported from Lawrenson et al. (2023)[1] meta-analysis. |
| SER difference v SVL at year 2 (D) |  |  |  |
| PPSL | 0 | N/A | Lawrenson et al. (2023)[1] meta-analysis, non-significant result. |
| MFSCL | 0.30 | 0.06; normal | Lawrenson et al. (2023)[1] meta-analysis |
| Orthokeratology | 0.51 | 0.09; normal | SER effect assumed proportionally equal to axial length effect; 50% reduction estimated from 0.56 change from baseline to year 2 with SVL and mean difference of -0.28 mm for orthokeratology versus SVL reported from Lawrenson et al. (2023)[1] meta-analysis. |
| Discontinuation due to adverse events with orthokeratology | 5% | N/A | Lawrenson et al. (2023)[1] systematic review |
| Age when myopia begins to stabilise (years) | 15.6 | 0.2; normal | COMET Group (2013)[2] |
| Age when myopia reaches its final level (years) | 20 | 2; normal | Assumption based on COMET Group (2013)[2], chosen to allow variation between age 16 and 24. |
| 10-year PSC cataract incidence for emmetropia | 4.3% | 80, 1,770; beta | Kanthan et al. (2014)[3] |
| Odds ratio (OR) PSC cataract |  |  | Haarman et al. (2020)[4] meta-analysis |
| -3 D < SER ≤ -0.5 D | 1.56 | 0.13; lognormal |  |
| -6 D < SER ≤ -3 D | 2.55 | 0.33; lognormal |  |
| SER ≤ -6 D | 4.55 | 1.30; lognormal |  |
| 10-year nuclear cataract incidence for emmetropia | 18.0% | 219, 996; beta | Kanthan et al. (2014)[3] |
| Odds ratio (OR) nuclear cataract |  |  | Haarman et al. (2020)[4] meta-analysis |
| -3 D < SER ≤ -0.5 D | 1.79 | 0.48; lognormal |  |
| -6 D < SER ≤ -3 D | 2.39 | 1.15; lognormal |  |
| SER ≤ -6 D | 2.87 | 1.10; lognormal |  |
| Prevalence glaucoma |  |  | Fricke et al. (2022)[5] citing Mitchell et al. (1999)[6] as data source; SE assumed to be 10% of the mean. |
| SER > -1 D | 0% | 0; N/A |  |
| -3D < SER ≤ -1 D | 2.7% | 0.0027; beta |  |
| SER ≤ -3 D | 2.9% | 0.0029; beta |  |
| Prevalence MMD |  |  | Fricke et al. (2022)[5] citing Vongphanit et al. (2002)[7] as source data; SE assumed to be 10% of the mean. |
| SER > -1 D | 0% | 0; N/A |  |
| -3 D < SER ≤ -1 D | 0.7% | 0.0007; beta |  |
| -5 D < SER ≤ -3 D | 3.0% | 0.0030; beta |  |
| -7 D < SER ≤ -5 D | 11.4% | 0.0114; beta |  |
| -9 D < SER ≤ -7 D | 28.6% | 0.0286; beta |  |
| SER ≤ -9 D | 52.4% | 0.0524; beta |  |
| Incidence retinal detachment |  |  | Fricke et al. (2022)[5] citing Flitcroft (2012)[8], Li (2003)[9], Mitry et al. (2010)[10], Ogawa & Tanaka (1988)[11], Polkinghorne & Craig (2004)[12], Wong et al. (1999)[13] as source data; SE assumed to be 10% of the mean.  Note incidence reported between ages 55 and 83 converted to annual probability within model. |
| SER > -3 D | 0% | 0; N/A |  |
| -6 D < SER ≤ -3 D | 0.09% | 0.0001; beta |  |
| -9 D < SER ≤ -6 D | 1.8% | 0.0018; beta |  |
| -15 D < SER ≤ -9 D | 28.6% | 0.0286; beta |  |
| SER ≤ -15 D | 74.6% | 0.0746; beta |  |
| Prevalence low-vision |  |  | Fricke et al. (2022)[5] modelled values for vision impairment, citing Bourne et al. (2017)[14], Flaxman et al. (2017)[15], Tideman et al. (2016)[16], Verhoeven et al. (2015)[17]; SE assumed to be 10% of the mean. |
| -3D < SER ≤ -0.5D, age <60 | 0% | 0; N/A |  |
| -6D < SER ≤ -3D, age <60 | 0.95% | 0.0010; beta |  |
| -10D < SER ≤ -6D, age <60 | 2.94% | 0.0029; beta |  |
| SER ≤ -10D, age <60 | 14.01% | 0.0014; beta |  |
| -3D < SER ≤ -0.5D, age ≥60 | 0% | 0; N/A |  |
| -6D < SER ≤ -3D, age ≥60 | 1.03% | 0.0010; beta |  |
| -10D < SER ≤ -6D, age ≥60 | 6.01% | 0.0061; beta |  |
| SER ≤ -10D, age ≥60 | 28.88% | 0.0289; beta |  |
| Hazard ratio death in low-vision state | 1.23 | 0.04; lognormal | Christ et al. 2008[18], value for visual impairment. |
| Age-dependent mortality |  |  | Office for National Statistics life-tables for Wales 2017-2019[19]; weighted by percentage male. |
| Participation in full-time education |  |  | Welsh Government (2022)[20] |
| Age 16 years | 73% | 26,133, 9,666; beta |  |
| Age 17 years | 67% | 23,315, 11,484; beta |  |
| Age 18 years | 54% | 18,467, 15,762; beta |  |
| SVL strategy costs |  |  |  |
| WGOS voucher SER > -6 D | £22 | N/A | Communication with Welsh Government; applied annually before age 16, then every two years for ages 16 to 18 years (Department of Health 2002[21]) subject to participation in full-time education. |
| WGOS voucher SER ≥ -6 D | £42 | N/A |  |
| WGOS sight test | £43 | N/A |  |
| PPSL strategy costs |  |  |  |
| Monthly payment | £44 | N/A | Estimated from prices of payment plans for MiYOSMART or Stellest lenses, fitting and follow-up published on the websites of private opticians as of January 2023.^a^ |
| WGOS sight test | N/A | N/A | Costs not applied to avoid double counting with monthly payment. |
| MFSCL strategy costs |  |  |  |
| Initiation cost | £100 | N/A | Estimated from prices of payment plans for MiSight or NaturalVue lenses, fitting and follow-up published on the websites of private opticians as of January 2023.^a^ |
| Monthly payment | £40 | N/A |  |
| WGOS voucher SER > -6 D | £22 | N/A | Assumed spectacles required as back-up. |
| WGOS voucher SER ≥ -6 D | £42 | N/A |  |
| WGOS sight test | N/A | N/A | Costs not applied to avoid double counting with monthly payment. |
| Orthokeratology strategy costs |  |  |  |
| Initiation cost | £200 | N/A | Estimated from prices of payment plans for lenses, fitting and follow-up published on the websites of private opticians as of January 2023.^a^ |
| Monthly payment | £40 | N/A |  |
| WGOS sight test | N/A | N/A | Costs not applied to avoid double counting with monthly payment. |
| Discontinuation due to adverse events | £5.36 | 0.59 | Assumed to be the cost of hypromellose eye drops and a paraffin-based eye ointment used to treat corneal staining (NHS Prescription Services 2023[22]). This assumption may overestimate the true cost because corneal staining may be resolved without treatment for many children. |
| Low vision health state cost (annual) | £7,959 | 796; gamma | Cost for severe vision impairment health state in NG81 (NICE 2017[23]), citing Meads & Hyde (2003)[24] inflated from £7,046.85 (2015/16 prices); SE assumed 10% of the mean. |
| MMD cost (annual) | £60 | 6; gamma | Assumed that 8% (Wong et al. 2014[25]) treated for myopic choroidal neovascularisation with ranibizumab (£537 for single dose, BNF (2023)[26]) and out-patient minor vitreous retinal procedure (BZ87A, NHS England (2022)[27]). |
| Glaucoma cost (annual) | £631 | 63; gamma | Cost for early glaucoma in NG81 (NICE 2017[23]), citing Traverso et al. (2005)[28] inflated from £559 (2015/16 prices). |
| Retinal detachment cost (event) | £1,708 | 171; gamma | NHS England (2022)[27]; non-elective minor (BZ87A), intermediate (BZ86B) or major CC score 0-1 (BZ84B) vitreous retinal procedures, weighted by activity. |
| PSC or nuclear cataract cost (event) | £1,741 | 174; gamma | NHS England (2022)[27]; phacoemulsification cataract extraction and lens implant, any CC score (BZ34A-C), weighted by activity. |
| Age-dependent utilities |  |  | Hernández Alava et al. (2022)[29]; EQ-5D in England general population; utility for children aged <16 assumed equal to utility at age 16, weighted by percentage male. |
| Utility decrements (weight) |  |  |  |
| Mild myopia | 0.005 | 3, 549; beta | WHO 2020[30]; assumed disability weight for mild VI. |
| Moderate myopia | 0.005 | 3, 549; beta | WHO 2020[30]; assumed disability weight for mild VI. |
| High myopia | 0.089 | 15, 152; beta | WHO 2020[30]; assumed disability weight for moderate VI. |
| MMD | 0.202 | 21, 84; beta | WHO 2020[30]; assumed average of disability weights for moderate and severe VI; applied instead of myopia decrements. |
| Low vision | 0.326 | 19, 39; beta | WHO 2020[30]; assumed average of disability weights for severe VI and blind. |
| Glaucoma | 0.061 | 29, 439; beta | Jampel (2001)[31]; TTO US population with glaucoma. |
| Utility decrement (absolute loss) |  |  |  |
| Retinal detachment event | 0.0325 | 97, 2,879; beta | 0.13 (Busbee et al. 2002[32]) assumed to last 3 months (NICE TA346, TA613, TA824); SE assumed 10% of mean. |
| PSC or nuclear cataract event | 0.037 | 96, 2,505; beta | 0.148 (Busbee et al. 2002[32]) assumed to last 3 months; SE assumed 10% of mean. |
| Discontinuation due to adverse events | 0 | N/A | Assumed no decrement because corneal staining is typically resolved within a few days. |
| ^a^ Costs were identified from online searches of optician websites. Published prices were identified for different products across 10 providers, typically small independent practices/chains or university optometry services. Base case inputs were populated using a mid-range option among those identified.  Abbreviations: BNF, British National Formulary; CC, complications and co-morbidities; D, dioptres; HR, hazard ratio; MFSCL, multifocal soft contact lenses; MMD, myopic macular degeneration; NG, national guideline: OR, odds ratio; PPSL, peripheral plus spectacle lenses; PSC, Posterior subcapsular; SE, standard error; SER, spherical equivalent refractive error; SVL, single-vision lenses; TA, technology appraisal; TTO, time trade-off; VI, visual impairment of distance vision; WGOS, Wales General Ophthalmic Service | | | |

Table A2: Scenario analysis descriptions and results

| Scenario description | Pairwise ICER versus SVL | | | Fully incremental outcome | |
| --- | --- | --- | --- | --- | --- |
|  | MFSCL | PPSL | OK | Efficiency frontier | Optimal |
| Base case | £8,367 | >£1M | £3,995 | SVL, OK | OK |
| NMA effects from Lawrenson et al. (2023)[1] applied and PPSL continued up to age 16 | £5,861 | £6,334 | £3,462 | SVL, OK | OK |
| Orthokeratology effects modelled based on relationships between SER progression and axial length progression from Jakobsen et al. (2021)[33] or Chen et al. (2023)[34] | £8,367 | >£1M | £3,996 to £4,410 | SVL, OK | OK |
| Extrapolation of 2-year average change in SER | £3,695 | >£1M | £1,981 | SVL, OK | OK |
| All strategies stopped after two years to reflect trial durations with no extrapolation | £4,531 | >£1M | £2,565 | SVL, OK | OK |
| All myopia controls stopped at age 13 years to reflect lower entry criteria in some trials | £6,042 | >£1M | £3,006 | SVL, OK | OK |
| MFSCL and orthokeratology continued for 16 to 18 year olds, irrespective of education | £10,240 | >£1M | £5,238 | SVL, OK | OK |
| 4.5 adverse events per 100 patient years with MFSCL (Cheng et al. 2020)[35]; cost £5.36 and no utility decrement per event | £8,372 | >£1M | £3,995 | SVL, OK | OK |
| 13.9 microbial keratitis events per 10,000 patient years with orthokeratology (Bullimore et al. 2013[36]); cost £4,211 (Moussa et al. 2021[37]) and 0.03 utility decrement (assumed 0.1 for 3 months from Arunga et al. 2019[38]) | £8,367 | >£1M | £4,057 | SVL, OK | OK |
| Orthokeratology adverse events resolved at no cost | £8,367 | >£1M | £3,995 | SVL, OK | OK |
| No discontinuation due to adverse events in any arm | £8,367 | >£1M | £3,958 | SVL, OK | OK |
| 50% discontinuation of orthokeratology in first cycle | £8,367 | >£1M | £4,674 | SVL, OK, MFSCL | OK |
| Lower and upper intervention cost scenarios | £4,585 to £13,855 | £774,258 to >£1M | £2,673 to £6,898 | SVL, OK | OK |
| One-off cost of cycloplegic refraction with SVL | £8,176 | >£1M | £3,890 | SVL, OK | OK |
| Ages at stabilisation and final myopia level sampled | £8,051 | >£1M | £4,000 | SVL, OK | OK |
| No progression modelled after age at stabilisation | £12,379 | >£1M | £7,260 | SVL, OK | OK |
| Progression continued to age 24 years | £8,059 | >£1M | £3,454 | SVL, OK | OK |
| Fast progressing cohort: -0.75 D annual progression, 6-monthly sight tests and optical vouchers until age of 16 years in SVL arm; no change to the effect inputs | £13,606 | £686,124 | £5,225 | SVL, OK | OK |
| Prevalence and mortality for low vision state informed by blindness data (Fricke et al. 2022[5] and Christ et al. 2008[18]) | £9,012 | >£1M | £4,627 | SVL, OK | OK |
| Long-term complications modelled from age 40 years | £7,474 | >£1M | £3,294 | SVL, OK | OK |
| WHO moderate VI disability weight applied to moderate myopia | £43,009 | £65,974 | £10,035 | SVL, OK | OK |
| Older WHO disability weights (Salomon et al. 2015[39]) applied | £23,681 | >£1M | £11,285 | SVL, OK | OK |
| Glaucoma utility decrement varied between 0.02 to 0.10 to reflect values applied to early and moderate glaucoma in NG81 (NICE 2017[23]) | £8,366 to £8,368 | >£1M | £3,995 to £3,996 | SVL, OK | OK |
| Cataract utility decrement of 0.08 applied to reflect decrement of 0.28 for 109 days (Hopkins et al. 2008[40]) | £8,358 | >£1M | £3,990 | SVL, OK | OK |
| Fixed baseline utility instead of age-dependent | £8,361 | >£1M | £3,993 | SVL, OK | OK |
| Multiplicative application of utilities | £9,650 | >£1M | £4,614 | SVL, OK | OK |
| Abbreviations: D, dioptres; ICER, incremental cost effectiveness ratio; M, million; MFSCL, multifocal soft contact lenses; NG, national guideline; NMA, network meta-analysis; OK, orthokeratology; PPSL, peripheral plus spectacle lenses; QALY, quality-adjusted life-year; SER, spherical equivalent refractive error; SVL, single-vision lenses; VI, visual impairment | | | | | |

Table A3: Budget impact model inputs

| **Input** | **Mean** | **Source and derivation** |
| --- | --- | --- |
| Population distribution by age |  | ONS (2022)[41]; mid-2021 estimates for Wales |
| Prevalence of myopia by age |  | Rudnicka et al. (2010)[42] and McCullough et al. (2016)[43]; prevalence at age 8 to 9 years assumed 2.65%; prevalence over age 14 to 15 years assumed 16.4%. May underestimate prevalence slightly as estimates come from White populations, which accounts for 94% of the Welsh population.[44] |
| 6 to 7 years | 1.9% |  |
| 10 to 11 years | 3.4% |  |
| 12 to 13 years | 16.4% |  |
| Strategy costs |  | From cost-utility analysis (Table 1) |
| Eligibility | 100% | Assumptions; will lead to overestimate of true budget impact |
| Uptake | 100% |  |
| Discontinuation due to adverse events | 0% |  |
| SER in first five years (D) | > -6 |  |
| Abbreviation: D, dioptres; ONS, Office for National Statistics; SER, spherical equivalent refractive error | | |

Table A3: Estimation of population size for budget impact analysis

| **Age (years)** | **ONS population estimate** | **Modelled population ^a^** | **Prevalence of myopia (%) ^b^** | **Prevalent cohort with myopia** |
| --- | --- | --- | --- | --- |
| 6 | 34,235 | 35,885 | 1.90% | 682 |
| 7 | 34,498 | 35,885 | 1.90% | 682 |
| 8 | 35,563 | 35,885 | 2.65% | 951 |
| 9 | 36,615 | 35,885 | 2.65% | 951 |
| 10 | 37,225 | 35,885 | 3.40% | 1,220 |
| 11 | 36,725 | 35,885 | 3.40% | 1,220 |
| 12 | 36,581 | 35,885 | 16.40% | 5,885 |
| 13 | 37,261 | 35,885 | 16.40% | 5,885 |
| 14 | 35,375 | 35,885 | 16.40% | 5,885 |
| 15 | 34,767 | 35,885 | 16.40% | 5,885 |
| Total |  |  |  | 29,247 |
| ^a^ Variation in reported population by age assumed not to be significant so averaged across ages bands  ^b^ Prevalence for ages 8 to 9 interpolated and prevalence assumed constant above age 13 in the absence of data  Abbreviation: ONS, Office for National Statistics; | | | | |


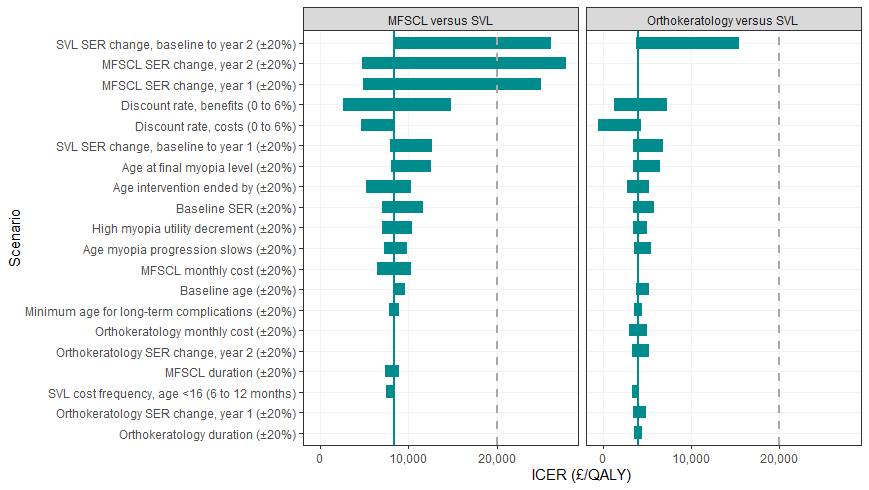
Figure A1: Most influential deterministic sensitivity analyses across pairwise comparisons of MFSCL or orthokeratology against SVL

References for additional file 1

1. Lawrenson JG, Shah R, Huntjens B, Downie LE, Virgili G, Dhakal R, et al. Interventions for myopia control in children: a living systematic review and network meta-analysis. Cochrane Database of Systematic Reviews. 2023;Issue 2:Art. No.: CD014758.

2. COMET Group. Myopia stabilization and associated factors among participants in the Correction of Myopia Evaluation Trial (COMET). Invest Ophthalmol Vis Sci. 2013;54(13):7871-83.

3. Kanthan GL, Mitchell P, Rochtchina E, Cumming RG, Wang JJ. Myopia and the long-term incidence of cataract and cataract surgery: the Blue Mountains Eye Study. Clinical & Experimental Ophthalmology. 2014;42(4):347-53.

4. Haarman AEG, Enthoven CA, Tideman JWL, Tedja MS, Verhoeven VJM, Klaver CCW. The complications of myopia: a review and meta-analysis. Invest Ophthalmol Vis Sci. 2020;61(4):49.

5. Fricke TR, Sankaridurg P, Naduvilath T, Resnikoff S, Tahhan N, He M, et al. Establishing a method to estimate the effect of antimyopia management options on lifetime cost of myopia. Br J Ophthalmol. 2023;107(8):1043.

6. Mitchell P, Hourihan F, Sandbach J, Wang JJ. The relationship between glaucoma and myopia: the Blue Mountains Eye Study. Ophthalmology. 1999;106(10):2010-5.

7. Vongphanit J, Mitchell P, Wang JJ. Prevalence and progression of myopic retinopathy in an older population. Ophthalmology. 2002;109(4):704-11.

8. Flitcroft DI. The complex interactions of retinal, optical and environmental factors in myopia aetiology. Progress in Retinal and Eye Research. 2012;31(6):622-60.

9. Li X. Incidence and epidemiological characteristics of rhegmatogenous retinal detachment in Beijing, China. Ophthalmology. 2003;110(12):2413-7.

10. Mitry D, Charteris DG, Fleck BW, Campbell H, Singh J. The epidemiology of rhegmatogenous retinal detachment: geographical variation and clinical associations. Br J Ophthalmol. 2010;94(6):678-84.

11. Ogawa A, Tanaka M. The relationship between refractive errors and retinal detachment--analysis of 1,166 retinal detachment cases. Japanese Journal of Ophthalmology. 1988;32(3):310-5.

12. Polkinghorne PJ, Craig JP. Northern New Zealand rhegmatogenous retinal detachment study: epidemiology and risk factors. Clinical & Experimental Ophthalmology. 2004;32(2):159-63.

13. Wong TY, Tielsch JM, Schein OD. Racial difference in the incidence of retinal detachment in Singapore. Archives of Ophthalmology. 1999;117(3):379-83.

14. Bourne RRA, Flaxman SR, Braithwaite T, Cicinelli MV, Das A, Jonas JB, et al. Magnitude, temporal trends, and projections of the global prevalence of blindness and distance and near vision impairment: a systematic review and meta-analysis. Lancet Global Health. 2017;5(9):e888-e97.

15. Flaxman SR, Bourne RRA, Resnikoff S, Ackland P, Braithwaite T, Cicinelli MV, et al. Global causes of blindness and distance vision impairment 1990-2020: a systematic review and meta-analysis. Lancet Global Health. 2017;5(12):e1221-e34.

16. Tideman JW, Snabel MC, Tedja MS, van Rijn GA, Wong KT, Kuijpers RW, et al. Association of axial length with risk of uncorrectable visual impairment for Europeans with myopia. JAMA Ophthalmology. 2016;134(12):1355-63.

17. Verhoeven VJ, Wong KT, Buitendijk GH, Hofman A, Vingerling JR, Klaver CC. Visual consequences of refractive errors in the general population. Ophthalmology. 2015;122(1):101-9.

18. Christ SL, Lee DJ, Lam BL, Zheng DD, Arheart KL. Assessment of the effect of visual impairment on mortality through multiple health pathways: structural equation modeling. Invest Ophthalmol Vis Sci. 2008;49(8):3318-23.

19. Office for National Statistics. National life tables: Wales 2021 [13 July 2022]. Available from: <https://www.ons.gov.uk/peoplepopulationandcommunity/birthsdeathsandmarriages/lifeexpectancies/datasets/nationallifetableswalesreferencetables>.

20. Welsh Government. Participation of young people in education and the labour market: 2020 and 2021 (provisional): Welsh Government; 2022 [16 Jan 2023]. Available from: <https://www.gov.wales/participation-young-people-education-and-labour-market-2020-and-2021-provisional-html#section-107349>.

21. Department of Health. Memorandum of understanding on the frequency of NHS sight tests: Department of Health; 2002 [16 Jan 2023]. Available from: <https://webarchive.nationalarchives.gov.uk/ukgwa/+/www.dh.gov.uk/en/Publicationsandstatistics/Lettersandcirculars/Dearcolleagueletters/DH_4002763>.

22. NHS Prescription Services. Electronic Drug Tariff: NHS Business Services Authority, Department of Health and Social Care; 2023 [06 Feb 2023]. Available from: <https://www.nhsbsa.nhs.uk/pharmacies-gp-practices-and-appliance-contractors/drug-tariff>.

23. NICE. Glaucoma: diagnosis and management [Original Publication Date: 01 Nov 2017; Last updated: 26 Jan 2022]: National Institute for Health and Care Excellence; 2022 [6 Feb 2023]. Available from: <https://www.nice.org.uk/guidance/ng81>.

24. Meads C, Hyde C. What is the cost of blindness? Br J Ophthalmol. 2003;87(10):1201-4.

25. Wong TY, Ferreira A, Hughes R, Carter G, Mitchell P. Epidemiology and disease burden of pathologic myopia and myopic choroidal neovascularization: an evidence-based systematic review. Am J Ophthalmol. 2014;157(1):9-25.e12.

26. BNF. Ranibizumab [specialist drug]: medicinal forms 2023 [05 July 2023]. Available from: <https://bnf.nice.org.uk/drugs/ranibizumab/medicinal-forms/>

27. NHS England. National Cost Collection for the NHS 2020/21: National Health Service; 2022 [11 Aug 2022]. Available from: <https://www.england.nhs.uk/costing-in-the-nhs/national-cost-collection/>.

28. Traverso CE, Walt JG, Kelly SP, Hommer AH, Bron AM, Denis P, et al. Direct costs of glaucoma and severity of the disease: a multinational long term study of resource utilisation in Europe. Br J Ophthalmol. 2005;89(10):1245-9.

29. Hernández Alava M, Pudney S, Wailoo A. Estimating EQ-5D by age and sex for the UK 2022 [25 Oct 2022]. Available from: <https://www.sheffield.ac.uk/nice-dsu/methods-development/estimating-eq-5d>.

30. WHO. WHO methods and data sources for global burden of disease estimates 2000-2019: World Health Organization; 2020 [20 Dec 2022]. Available from: <https://cdn.who.int/media/docs/default-source/gho-documents/global-health-estimates/ghe2019_daly-methods.pdf?sfvrsn=31b25009_7>.

31. Jampel HD. Glaucoma patients' assessment of their visual function and quality of life. Transactions of the American Ophthalmological Society. 2001;99:301-17.

32. Busbee BG, Brown MM, Brown GC, Sharma S. Incremental cost-effectiveness of initial cataract surgery. Ophthalmology. 2002;109(3):606-12.

33. Jakobsen TM, Gehr NL, Møller F. Correlation between change in cycloplegic spherical equivalent refractive error and change in axial length in Danish children aged 6 to 12 year. Acta Ophthalmologica. 2021;99(7):e1249-e50.

34. Chen Z, Zhang Z, Xue F, Zhou J, Zeng L, Qu X, et al. The relationship between myopia progression and axial elongation in children wearing orthokeratology contact lenses. Contact lens anterior eye. 2023;46(1):Article 101517.

35. Cheng X, Brennan NA, Toubouti Y, Greenaway NL. Safety of soft contact lenses in children: retrospective review of six randomized controlled trials of myopia control. Acta Ophthalmologica. 2020;98(3):e346-e51.

36. Bullimore MA, Sinnott LT, Jones-Jordan LA. The risk of microbial keratitis with overnight corneal reshaping lenses. Optom Vis Sci. 2013;90(9):937-44.

37. Moussa G, Hodson J, Gooch N, Virdee J, Penaloza C, Kigozi J, et al. Calculating the economic burden of presumed microbial keratitis admissions at a tertiary referral centre in the UK. Eye. 2021;35(8):2146-54.

38. Arunga S, Wiafe G, Habtamu E, Onyango J, Gichuhi S, Leck A, et al. The impact of microbial keratitis on quality of life in Uganda. BMJ Open Ophthalmology. 2019;4(1):e000351.

39. Salomon JA, Haagsma JA, Davis A, de Noordhout CM, Polinder S, Havelaar AH, et al. Disability weights for the Global Burden of Disease 2013 study. Lancet Global Health. 2015;3(11):e712-23.

40. Hopkins RB, Tarride JE, Bowen J, Blackhouse G, O'Reilly D, Campbell K, et al. Cost-effectiveness of reducing wait times for cataract surgery in Ontario. Canadian Journal of Ophthalmology. 2008;43(2):213-7.

41. Office for National Statistics. Estimates of the population for the UK, England, Wales, Scotland and Northern Ireland. Mid-2021 edition of this dataset. 2022 [12 Jan 2023]. Available from: <https://www.ons.gov.uk/peoplepopulationandcommunity/populationandmigration/populationestimates/datasets/populationestimatesforukenglandandwalesscotlandandnorthernireland>.

42. Rudnicka AR, Owen CG, Nightingale CM, Cook DG, Whincup PH. Ethnic differences in the prevalence of myopia and ocular biometry in 10- and 11-year-old children: the Child Heart and Health Study in England (CHASE). Invest Ophthalmol Vis Sci. 2010;51(12):6270-6.

43. McCullough SJ, O'Donoghue L, Saunders KJ. Six year refractive change among white children and young adults: evidence for significant increase in myopia among white UK children. PloS one. 2016;11(1):e0146332.

44. Welsh Government. Ethnic group, national identity, language and religion in Wales (Census 2021) 2022 [06 Nov 2023]. Available from: <https://www.gov.wales/ethnic-group-national-identity-language-and-religion-wales-census-2021-html>.
